# A novel approach to studying human orogastric transit with an ingestible bionic device. An early feasibility study

**DOI:** 10.1088/1361-6579/ae52a2

**Published:** 2026-03-27

**Authors:** D Sun, G Barron Del Solar, X Guo, F J Moawad, J E Pandolfino, H Gregersen

**Affiliations:** 1 California Medical Innovations Institute, San Diego, CA, United States of America; 2 Chongqing Engineering Research Center of Medical Electronics and Information Technology, Chongqing University of Posts and Telecommunications, Chongqing, People’s Republic of China; 3 Scripps Clinic, Division of Gastroenterology, La Jolla, CA, United States of America; 4 Northwestern University, Department of Gastroenterology and Hepatology, Feinberg School of Medicine, Chicago, IL, United States of America

**Keywords:** esophagus, transit, swallowing, bionic, axial pressure, motility

## Abstract

**Objective.:**

A novel bionic esophageal device was developed to assess human swallowing function and orogastric transit, aiming ultimately to improve diagnostics for dysphagia. This miniaturized, tethered device records axial pressures, orientation, and acceleration during esophageal transit, thereby providing a dynamic view of the swallowing process.

**Approach.:**

In first-in-human feasibility tests, two healthy volunteers safely swallowed the device repeatedly in seated and supine positions.

**Main Results.:**

The system produced transit and pressure profiles comparable to existing technologies, with prolonged transit times observed in the supine position, e.g. transit time in seated position was median 6 s (6–23) and in the supine posture median 233 s [142–317]).

**Significance.:**

These findings support the potential of this bionic device for studying esophageal motility in physiological studies as well as pathological conditions in dysphagia patients, and for future translation to untethered capsule systems capable of full gastrointestinal transit analysis.

## 1. Background

Bionic technology refers to the integration of advanced mechanical, electronic, and robotic systems with the human body to restore, replace, or enhance biological functions, or for monitoring of physiological data from the body in real time. Bionic or biomimetic technologies have increasingly entered gastroenterological research and diagnostics. Ingestible capsules such as *PillCam* and multimodal measuring devices like *Fecobionics* have shown the feasibility of integrating physiological sensing with natural organ motion (Basar et al 2012, Gregersen et al 2023, Sun et al 2025). Dysphagia, a symptom complex defined by delayed or impaired swallowing, affects up to one in six adults, with prevalence increasing markedly after age 50 (Cha et al 2023, Sanagapalli et al 2023, Choi et al 2024). While oropharyngeal dysphagia is frequently neurological in origin, esophageal dysphagia arises from structural or motility disorders such as strictures, ineffective esophageal motility, achalasia, and esophagogastric junction outflow obstruction (EGJOO).

Traditional investigations—high-resolution manometry (HRM), impedance-HRM, contrast barium swallow, and EndoFLIP—have defined modern esophageal diagnostics (Carlson et al 2018, 2021, Clarke et al 2020). However, these are largely catheter-based, require specialized equipment, and offer limited dynamic data on bolus motion and orientation. There remains a clinical need for non-catheterized, physiologically integrated testing.

Building on prior animal and benchtop studies of biomimetic esophageal and anorectal models (Gregersen et al 2023, Sun et al 2025), we developed a tethered bionic esophageal device to capture real-time data on axial pressures, orientation, and motion during swallowing. Preliminary trials evaluated its safety, feasibility, and correspondence with known physiological parameters. We hypothesized that (1) measured esophageal pressures and transit velocities would fall within ranges reported for conventional tests; and (2) supine swallowing would produce significantly delayed transit, simulating a dysphagic pattern (Nguyen et al 1997a). The long-term vision is to apply both tethered and untethered versions to comprehensive studies of orogastric motility.

## 2. Methods

### 2.1. Device design

**Figure 1.**
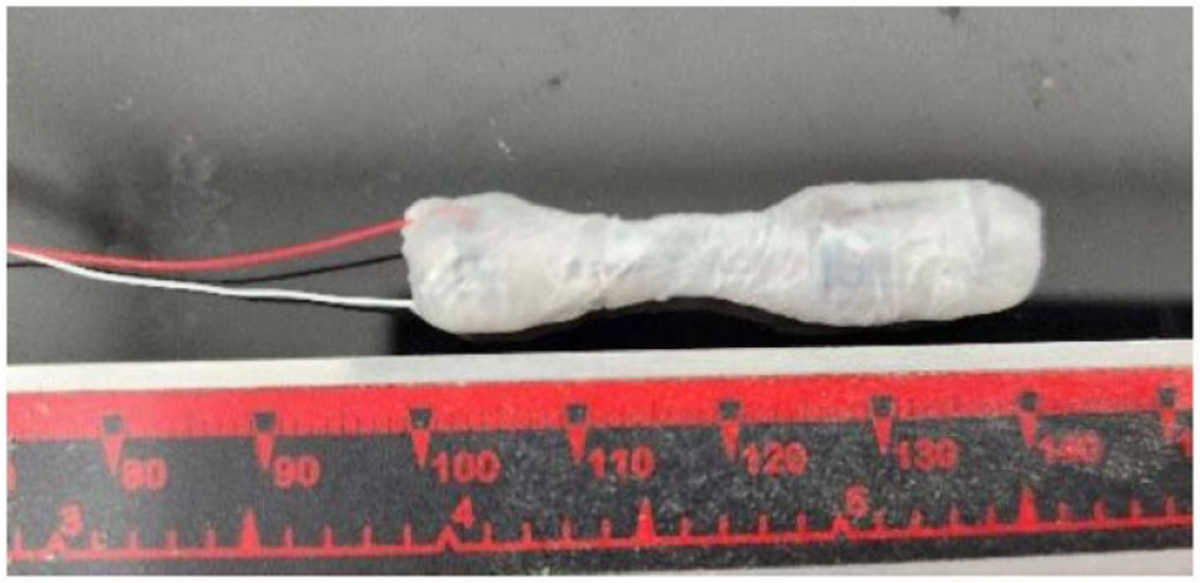
Photo of the 4.2 cm hourglass-shaped swallowable device. The mid-section is flexible. The system measures pressure, orientation, and acceleration in real time.

Two swallowable devices of 5.0 cm and 4.2 cm length were constructed (figure 1). The selection of length was based on the swallow experiments with gummies of different sizes and consistency that the subjects aimed to swallow without bending them in the mouth (supplementary material). Each device had an hourglass profile for flexibility, with maximum diameters of 8.5 mm at both ends. Pressure sensors and miniature motion processing units incorporating accelerometers, gyroscopes, and magnetometers were placed proximally (tether end) and distally. Data were sampled at 50 Hz, providing high-resolution temporal profiles of motion and pressure. A wireless transmitter enabled real-time signal transmission to an external receiver. The tether, composed of a fine, flexible wire leading to the external power supply, also permitted retrieval of the device.

### 2.2. Study design and participants

Two healthy adult volunteers (female 42 yrs, 50 kg; male 62 yrs, 85 kg) participated. Both denied gastrointestinal symptoms such as heartburn or dysphagia. The study involved multiple swallow trials in seated and lying positions, following established safety criteria for orogastric studies (Lamki 1985, Nguyen et al 1997a). Ethical approval (Advarra IRB: CALM-CLIN-2025–01 (Pro00087795) and informed consent were obtained in accordance with institutional review standards in accordance with the Declaration of Helsinki.

To simulate conditions of slowed esophageal clearance, supine swallows were performed without local anesthesia, facilitated by co-swallowing water or small banana pieces. Following gastric entry, the device was retracted to mid-esophagus for stationary measurements during dry, wet, and banana swallows. The principal outcome measures were **transit duration, pressure amplitudes**, and **bending/orientation dynamics**. Given the exploratory nature of the study, formal statistical comparisons were not undertaken; results are presented as medians with interquartile ranges.

### 2.3. Data analysis

Negative pressure events identified the oral initiation phase, followed by esophageal peristaltic contractions visible in the pressure traces. Orientation was estimated using a quaternion-based Madgwick algorithm combining accelerometer, gyroscope and magnetometer data. The estimated quaternion provided the transformation from sensor to earth frame. Gravitational acceleration was identified and subtracted to obtain linear acceleration prior to integration. The gravity-compensated linear acceleration was smoothed using the Savitzky–Golay filter (*λ* = 0.2) to reduce high-frequency sensor noise while preserving transient peristaltic acceleration peaks. Velocity was obtained by numerical integration, and displacement by subsequent integration of velocity. During prolonged low-velocity supine transit, quasistatic periods were used as zero-velocity reference segments to limit cumulative drift. For total travelled distance, the use of the stationary gastric phase as a zero-velocity reference constrains cumulative drift, and the expected overall distance uncertainty was estimated to be ±3–5 cm. Safety assessment was based on subject reports and observation of any adverse or device-related effects.

## 3. Results

Both subjects successfully completed the protocol, performing five swallows in each posture. The 4.2 cm device was favored after one episode of u-bending with the 5.0 cm prototype. Swallowing was easiest when aided by a small banana bolus. No anesthesia was needed, and both participants tolerated the device well.

### 3.1. Oropharyngeal and esophageal pressures

**Figure 2.**
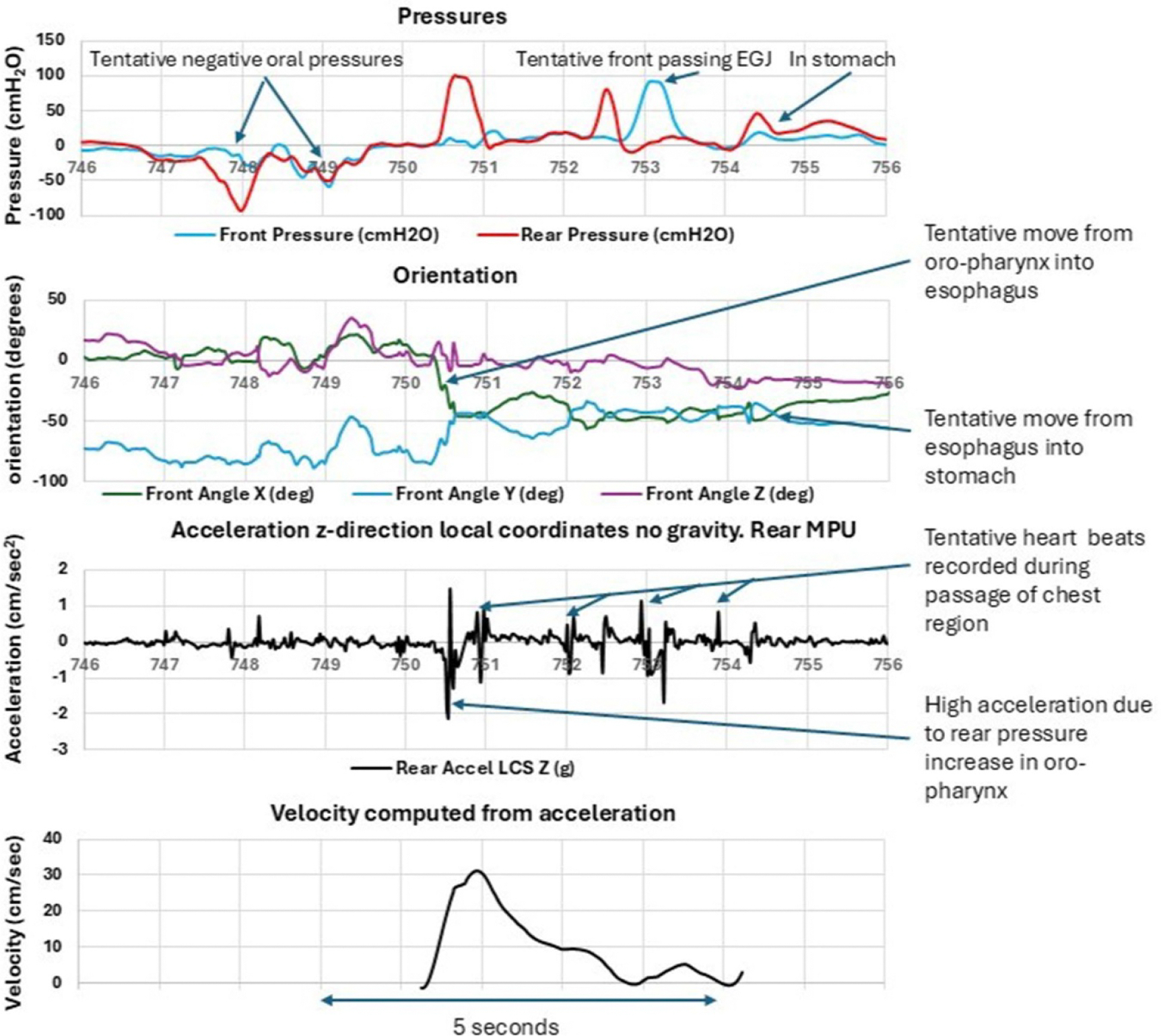
Representative data from a swallow in seated position. From top to bottom: recorded pressures, orientation (three axes), acceleration, and computed velocity. Transit from mouth to stomach occurred within ≈ 6 s. Negative pressure marked oral initiation, followed by a rear-channel contraction and orientation shift at esophageal entry. Acceleration peaks correspond to peristaltic propulsion, and the second pressure peak (≈252–253 s) likely marks passage through the lower esophageal sphincter. Integration of acceleration yielded velocities up to 30 cm s^−1^ and an estimated travel distance of 35 cm. Regular minor fluctuations (~1 Hz) during thoracic transit may correspond to cardiac pulsations.

**Figure 3.**
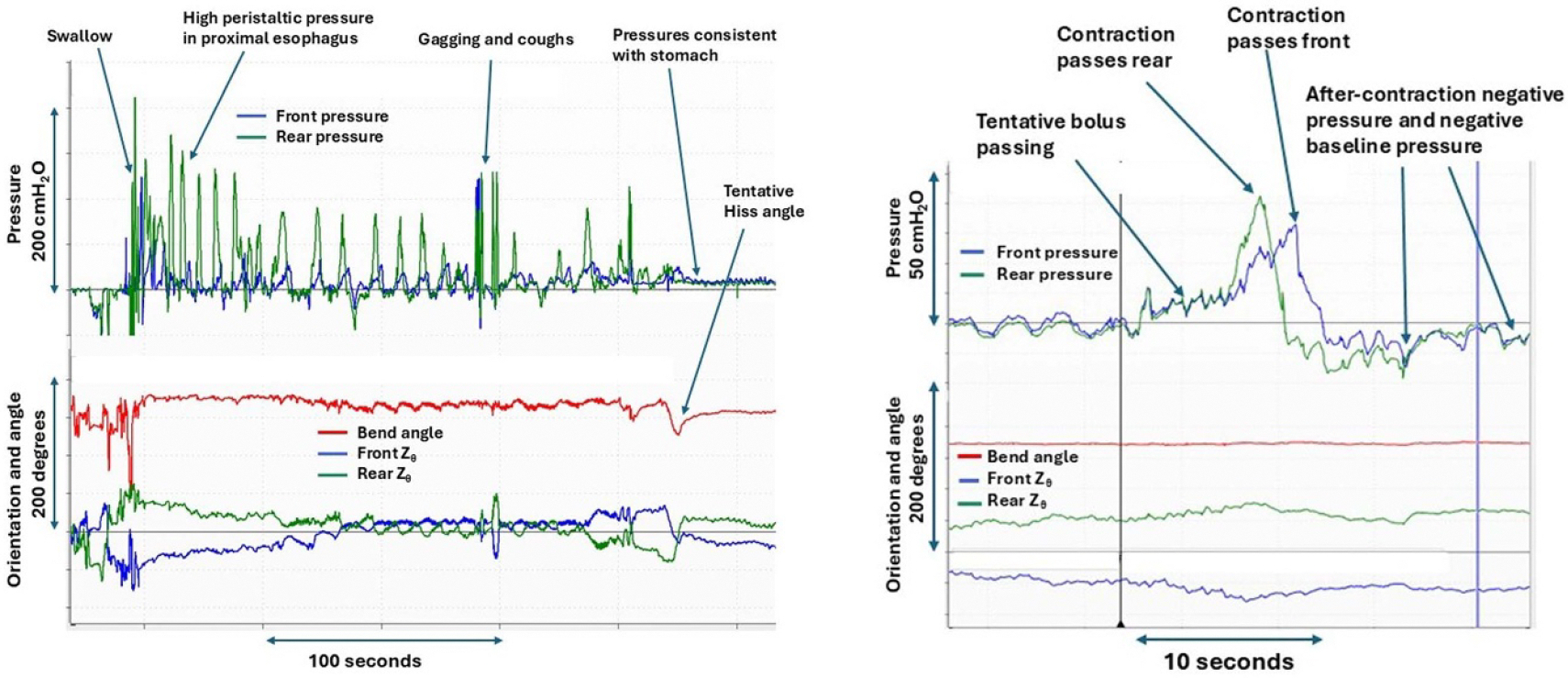
Left: swallow performed in supine position showing prolonged transit (~230 s) relative to seated posture. Toward the end (~730 s), a bend likely represents the Hiss angle. Right: stationary mid-esophageal recording during banana swallow showing baseline pressure rise, rear and front pressure peaks, and subsequent negative after-contraction deflections.

During the oral phase, characteristic short-lasting negative pressure peaks were recorded (seated = −141 cmH_2_O [−162 to −125]; supine = −150 cmH_2_O [−182 to −96]). In the seated position, esophageal transit time was brief, median 6 s (6–23), whereas in the supine posture, transit was markedly prolonged (median = 233 s [142–317]) (figures 2 and 3). Peristaltic contractions produced the highest amplitudes proximally in esophagus, consistent with manometric findings (Nguyen et al 1999, Carlson et al 2021). The maximum rear, front, and delta (rear—front) pressures were, respectively for seated position 195 (163–222), 99 (64–164), 200 (165–202) cmH_2_O, and for supine position 165 (143–196), 77 (41–110), 173 (135–199) cmH_2_O.

### 3.2. Dynamic orientation and acceleration

Orientation and acceleration data indicated the transitions from oropharynx to esophagus and from esophagus to stomach. Integration of the acceleration signal yielded velocity profiles (figure 2) consistent with published ranges (Kern et al 1996, Nguyen et al 1999). The estimated distance traversed from the oral cavity to stomach was approximately 35 cm. Fluctuations in acceleration at 1 Hz during thoracic transit may tentatively correspond to cardiac motion, a phenomenon warranting further investigation (figure 2).

### 3.3. Gastric and stationary measurements

Intragastric placement yielded stable positive pressure baselines that rose during leg raises (≈+50 cmH_2_O) and deep inspiration. When retracted to mid-esophagus, leg raises induced smaller increases (≈+30 cmH_2_O) and inspiration caused negative excursions, confirming correct placement. Dry, wet, and banana swallows elicited instantaneous rear pressure peaks of 70–100 cmH_2_O, with bolusinduced pre-contraction pressure rises most evident during banana swallows (figure 3). No adverse or technical events were recorded. Minor transient gagging occurred in one subject but was rated as less uncomfortable than prior HRM experience.

## 4. Discussion

This first-in-human evaluation demonstrates that a miniaturized bionic esophageal device can be swallowed safely, generate physiologically meaningful data, and reproduce expected motility patterns observed with standard manometric and impedance-based techniques. Despite the minimal number of participants, the consistency of results across multiple swallows suggests that the system accurately captures dynamic events associated with orogastric transit.

### 4.1. Physiological correspondence with established methods

The pressure and velocity data obtained were comparable to prior reports using HRM, impedance-HRM, and radiographic transit studies. Negative oral pressures during the initiation phase, followed by high-amplitude proximal contractions and longer distal transit times, correspond well to reported swallowing mechanics (McConnel 1988, Nguyen et al 1997a, Carlson et al 2021). The finding of markedly prolonged transit in the supine position aligns with radionuclide and impedance-based observations that gravitational effects facilitate esophageal clearance (Lamki 1985, Choi et al 2024).

Importantly, the simultaneous measurement of axial pressure, orientation, and acceleration provides an integrated physiological view that existing tools cannot achieve. Conventional HRM measures luminal pressure but not the device’s spatial behavior or bolus kinematics. The new bionic platform therefore bridges mechanical and functional data streams, allowing direct interpretation of propulsive versus resistive forces. This distinction is relevant for identifying esophageal motility phenotypes such as EGJOO and spastic disorders.

### 4.2. Advantages and clinical translation potential

The simplicity of the system offers several translational benefits. The device connects wirelessly to a compact external receiver and can be controlled via a laptop or tablet application, requiring no large manometry station or radiographic equipment, i.e. no capital equipment. It is therefore suitable for decentralized or point-of-care motility screening, extending diagnostic capability beyond tertiary laboratories. Because the tethered configuration permits safe retrieval, it can be used even in patients with mild esophageal narrowing, though pre-endoscopic assessment is recommended. However, at least at this point, it is not recommended for assessment of oropharyngeal dysphagia. Caution should be exercised in patients with severe strictures and high aspiration risk.

Beyond dysphagia assessment, the proposed bionic approach could facilitate quantitative evaluation of other upper GI conditions—such as post-fundoplication dysmotility, scleroderma-associated hypomotility, or postoperative recovery following myotomy. Integration with impedance or pH sensors, or coupling to optical or acoustic flow tracking, could further enhance diagnostic specificity (Ravinder and Tan et al 2022). Dysphagia affects both men and women and it is well-known that gender differences impact on the mechanisms, e.g. men has longer esophagi, but women often have higher sphincter pressure and lower pain threshold.

### 4.3. Limitations and safety considerations

This feasibility study was limited by its small sample size and the absence of comparator data obtained concurrently with HRM or barium swallow. These were intentionally omitted to reduce procedural burden in early human trials. Future studies should evaluate reproducibility, inter-subject variability, and sensor calibration against gold-standard technologies. The accuracy of velocity estimation derived from acceleration integration also requires validation with fluoroscopic or impedance-based flow data (Steele and Miller 2021). However, velocity, transit time and pressure amplitudes were in the same range as published data (Kern et al 1996, Nguyen et al 1999, Carlson et al 2021) but direct comparative data will be needed in future trials. Parameters may differ due to differences in experimental conditions, e.g. a stationary pressure catheter will measure the radial contraction pressure whereas the swallowable device likely will measure the axial driving pressure and fluid resistive pressures during esophageal passage.

Safety was satisfactory, with no adverse events, but broader testing will be needed to assess device tolerance across patient populations, particularly those prone to gagging or chest discomfort. While no complications occurred, the tether introduces a minor gagging risk, and care must be taken during insertion to prevent airway stimulation. Should tether rupture occur, the flexible, biocompatible body is designed to transit naturally through the gastrointestinal tract. The device’s short length and compliant design minimize perforation risk compared with larger, stiffer foreign bodies. The potential for rare airway interference remains theoretical but must be monitored carefully in dysphagia patients. In future patient studies, airway protection protocols should be standardized, particularly in those with known aspiration risk. Comparison with HRM and barium swallow outcomes will be necessary to establish diagnostic equivalence and clinical validity.

### 4.4. Future directions

Next-phase development includes testing in dysphagia subtypes—achalasia types I–III, EGJOO, hypertensive and spastic esophagus—where combined pressure-orientation patterns may yield unique diagnostic signatures. Complex spatiotemporal behaviors such as corkscrew contractions could be recognized directly by characteristic oscillations in the pressure and gyroscope data. Integration with impedance measurement or expandable bag sensors could enable bolus flow and compliance assessments, bridging toward a comprehensive ‘esophageal Fecobionics’ paradigm (Sun et al 2025).

An eventual untethered version, similar to smart motility capsules, could permit continuous monitoring from esophagus to colon (Park et al 2023). Such devices would, however, be contraindicated in patients with strictures or severe dysmotility until robust safety data exist. The tethered system remains the most controllable and ethically acceptable configuration for early patient studies.

## 5. Conclusion

This first demonstration of a swallowable, tethered bionic device for orogastric transit confirms the feasibility of safely acquiring simultaneous pressure, motion, and orientation data *in vivo*. The results reproduce known physiological parameters and highlight the capability to differentiate between positional and dynamic effects on swallowing. The approach complements traditional manometry and fluoroscopy by offering integrated, multidimensional motility assessment without anesthesia or radiation.

With further refinement and validation, bionic esophageal devices could become practical tools for motility screening and longitudinal monitoring in both research and clinical settings. The technology aligns with the broader movement toward minimally invasive, sensor-based physiological diagnostics in gastroenterology.

## Supplementary Material

Suppl material

Supplementary material available at https://doi.org/10.1088/1361-6579/ae52a2/data1.

Supplementary material for this article is available online

## Data Availability

All data that support the findings of this study are included within the article (and any supplementary information files).
