# Supplementary material for: A novel approach to studying human orogastric transit with an ingestible bionic device. An early feasibility study
Source: Physiol Meas. Author manuscript; Available in PMC 2026 May 22. (PMC13197135; doi:10.1088/1361-6579/ae52a2)
Supplement: Suppl material [file NIHMS2169839-supplement-Suppl_material.docx]

**Background with preliminary data**

**Animal experiments**

The idea for development of a swallowable device was conceived from the successful development of the anorectal wireless Fecobionics device. Though the 10cm-long and 10mm-wide Fecobionics device is way too big for a human to swallow, we used it in controlled experiments in a lightly sedated 60kg pig. When successful swallows were made by the pig (n=4), the device passed to the stomach in 4-6 seconds where after it recorded from the stomach for 1-2hrs until the batteries were drained. The pig expelled all devices after 24-72 hours and showed no signs of intestinal obstruction.

**Gummy swallowing experiments in humans**

More than 200 swallowing experiments were done in five subjects with gummies of various sizes, consistency, and surface smoothness. If the gummies were bendable and had a smooth surface, up to 10cm-long and 8mm-diameter gummies (less than 5ml volume) were easily swallowable if the subjects were allowed to bend them in the mouth. Adults can normally swallow 20-30mL food volumes without trouble. The largest gummies only had a volume of 5mL, i.e., less than 25% of max tolerable volumes swallowed by humans. We assumed that the gummies, simulating the device, would un-bend when entering the pharynx and esophagus.

**Preliminary device swallow attempts in humans**

Miniaturized devices first with length of 7cm and 7mm diameter could not be swallowed straight. Reduction of the length first to 6cm and then to 5.0cm and changing the shape slightly to hourglass shape with up to 9mm diameter at the ends gave hope that such devices could be swallowed by humans. The device must be swallowed straight rather than U-bend to produce meaningful data, i.e., the pressure sensors must point in opposite axial directions (in the direction of the trajectory) as well as analysis based on the gyroscopes and accelerometer data depend on correct configuration.
